# Protocol for the *3HP Options Trial*: a hybrid type 3 implementation-effectiveness randomized trial of delivery strategies for short-course tuberculosis preventive therapy among people living with HIV in Uganda

**DOI:** 10.1186/s13012-020-01025-8

**Published:** 2020-08-12

**Authors:** Jillian L. Kadota, Allan Musinguzi, Juliet Nabunje, Fred Welishe, Jackie L. Ssemata, Opira Bishop, Christopher A. Berger, Devika Patel, Amanda Sammann, Anne Katahoire, Payam Nahid, Robert Belknap, Patrick P. J. Phillips, Jennifer Namusobya, Moses Kamya, Margaret A. Handley, Noah Kiwanuka, Achilles Katamba, David Dowdy, Fred C. Semitala, Adithya Cattamanchi

**Affiliations:** 1 Division of Pulmonary and Critical Care Medicine and Center for Tuberculosis, University of California San Francisco, San Francisco, CA USA; 2 Infectious Diseases Research Collaboration, Kampala, Uganda; 3 Makerere University Joint AIDS Program, Kampala, Uganda; 4 Department of Surgery, Zuckerberg San Francisco General Hospital, University of California San Francisco, San Francisco, CA USA; 5 Child Health and Development Centre, Makerere University, Kampala, Uganda; 6 Denver Health and Hospital Authority, Denver, CO USA; 7 Division of Infectious Diseases, Department of Medicine, University of Colorado, Denver, CO USA; 8 University Research Company, Center for Human Services, Department of Defense HIV/AIDS Prevention Program (URC-DHAPP), Kampala, Uganda; 9 Department of Medicine, Makerere University College of Health Sciences, Kampala, Uganda; 10 Center for Vulnerable Populations at Zuckerberg San Francisco General Hospital and Trauma Center, University of California, San Francisco, San Francisco, CA USA; 11 Department of Epidemiology and Biostatistics, University of California, San Francisco, San Francisco, CA USA; 12 Department of Epidemiology and Biostatistics, School of Public Health, Makerere University, Kampala, Uganda; 13 Uganda Tuberculosis Implementation Research Consortium, Kampala, Uganda; 14 Department of Epidemiology, Johns Hopkins Bloomberg School of Public Health, Baltimore, MD USA; 15 Mulago- ISS Clinic, Old Mulago Hill Road, New Mulago Hospital Complex, P.O Box 7051, Kampala, Uganda

**Keywords:** Effectiveness-implementation hybrid, Rifapentine, Isoniazid, Tuberculosis preventive therapy, HIV/AIDS, Person-centered care, Patient choice, Preference trials

## Abstract

**Background:**

Recently, a 3-month (12-dose) regimen of weekly isoniazid and rifapentine (3HP) was recommended by the World Health Organization for the prevention of tuberculosis (TB) among people living with HIV (PLHIV) on common antiretroviral therapy regimens. The best approach to delivering 3HP to PLHIV remains uncertain.

**Methods:**

We developed a three-armed randomized trial assessing optimized strategies for delivering 3HP to PLHIV. The trial will be conducted at the Mulago Immune Suppression Syndrome (i.e., HIV/AIDS) clinic in Kampala, Uganda. We plan to recruit 1656 PLHIV, randomized 1:1 to each of the three arms (552 per arm). Using a hybrid type 3 effectiveness-implementation design, this pragmatic trial aims to (1) compare the acceptance and completion of 3HP among PLHIV under three delivery strategies: directly observed therapy (DOT), self-administered therapy (SAT), and informed patient choice of either DOT or SAT (with the assistance of a decision aid); (2) to identify processes and contextual factors that influence the acceptance and completion of 3HP under each delivery strategy; and (3) to estimate the costs and compare the cost-effectiveness of three strategies for delivering 3HP. The three delivery strategies were each optimized to address key barriers to 3HP completion using a theory-informed approach. We hypothesize that high levels of treatment acceptance and completion can be achieved among PLHIV in sub-Saharan Africa and that offering PLHIV an informed choice between the optimized DOT and SAT delivery strategies will result in greater acceptance and completion of 3HP. The design and planned evaluation of the delivery strategies were guided by the use of implementation science conceptual frameworks.

**Discussion:**

3HP—one of the most promising interventions for TB prevention—will not be scaled up unless it can be delivered in a patient-centered fashion. We highlight shared decision-making as a key element of our trial design and theorize that offering PLHIV an informed choice between optimized delivery strategies will facilitate the highest levels of treatment acceptance and completion.

**Trial registration:**

ClinicalTrials.gov: NCT03934931; Registered 2 May 2019.

Contributions to the literature

- We describe the design of a trial with a primary focus on comparing the uptake of short-course tuberculosis preventive therapy for people living with HIV (PLHIV)—an evidence-based intervention—under three delivery strategies optimized to overcome patient barriers to treatment adherence and completion. Informed patient choice of delivery strategy is highlighted as a key study design element.
- Study findings will demonstrate whether tuberculosis preventive therapy can be delivered as part of routine HIV/AIDS care in a manner that results in high levels of uptake and provide a comprehensive evaluation of different delivery strategies needed to inform scale-up in high HIV/TB burden settings.

## Background

Tuberculosis (TB) preventive therapy (TPT) is critical to reducing TB burden among people living with HIV (PLHIV). TPT, which has traditionally consisted of 6–9 months of daily isoniazid (INH) [1], reduces TB incidence by 30–50% and the risk of severe illness or death by 35% [2]. To date, however, uptake of TPT has been poor among PLHIV in high-burden countries. Globally, fewer than one million PLHIV were reported to have started TPT in 2017, and coverage among PLHIV ranged from 1 to 53% in 15 high TB/HIV burden countries [3]. Among those who initiate daily isoniazid treatment, common individual and clinic-level barriers to treatment completion such as stigma, concerns about toxicity and length of treatment, transport costs, and poor relationships with health care providers contribute to suboptimal completion rates [4–6]. The proportion lost before completing treatment increases with each additional month of therapy [7] and averages 52% in low- and middle-income countries (LMIC) [8].

In recent randomized trials, a 3-month (12-dose) regimen of weekly INH and rifapentine (RPT) (3HP) had equivalent efficacy, better tolerability, and higher completion relative to 9 months of daily INH [9, 10]. 3HP is compatible with common ART regimens used in many high-burden countries [10–13]. Thus, the World Health Organization’s (WHO) updated guidelines from 2018 recommend 3HP as an option for TB prevention among PLHIV.

While the potential of this shorter and safer regimen is promising, the best approach for delivering 3HP to PLHIV is unclear. 3HP was originally studied using directly observed therapy (DOT), which provides a regular opportunity to monitor patients for side effects and signs of toxicity. DOT also affords more frequent interactions with healthcare providers [14]. In contrast, DOT requires weekly clinic or field visits which can be prohibitively expensive and time consuming for patients and providers. While there is less opportunity for direct patient monitoring, self-administered therapy (SAT) is an attractive alternative that allows for greater patient autonomy and overcomes many of the challenges faced by patients on DOT. A recent multi-center trial that randomized patients to SAT versus DOT found SAT to be inferior to DOT (74–76% vs. 87% completion rates, respectively) [15]. Of the participating sites, SAT performed worst in South Africa (the only African site), with treatment completion of 37%. However, in that trial, neither the DOT nor SAT delivery strategies were specifically designed to address key barriers to treatment completion. In addition, patients may have a clear preference for DOT or SAT, and these preferences may influence adherence [15].

Innovative approaches to promoting and monitoring adherence to TB medications are now available [16] but have not been evaluated in the context of TPT. For example, 99DOTS (Everwell Health Solutions, India) is a novel, simple, and low-cost ($4–6/patient at scale) technology whereby medications are packaged alongside hidden toll-free phone numbers that are revealed when each dose is unpackaged, enabling patients to make toll-free calls to confirm medication dosing. Clinic staff can remotely access patient adherence data through a web dashboard and mobile phone application. Interactive voice response (IVR) reminders, check-in phone calls, and two-way messaging are also core features of the platform that enable real-time identification of patients who miss doses for further follow-up and monitoring of potential side effects. Thus, digital adherence technologies address well-known patient-level barriers to treatment adherence including lack of time and transportation to regularly attend clinic [17, 18].

Trials of theory-informed approaches to facilitate treatment adherence and completion are urgently needed in order to realize the potential of 3HP. No previous study has (1) evaluated whether high levels of 3HP acceptance and completion can be achieved—by DOT or SAT—in the context of routine HIV/AIDS care in sub-Saharan Africa, (2) adopted a theory-informed approach to optimizing 3HP delivery through treatment facilitation, or (3) identified the most effective and cost-effective 3HP delivery strategy for high-burden settings. In order to address this gap, the objectives of the *3HP Options Trial* are threefold:

1. To compare the acceptance and completion of 3HP under three delivery strategies (facilitated DOT, facilitated SAT, or informed patient choice between facilitated DOT and SAT);
2. To identify processes and contextual factors that influence the acceptance and completion of 3HP under each delivery strategy; and
3. To estimate the costs and compare the cost-effectiveness of the three strategies for delivering 3HP.

### Conceptual frameworks informing the 3HP Options Trial

Our trial design and 3HP delivery strategies were informed by a variety of tools, theories, and conceptual frameworks. Overall trial design was guided by hybrid effectiveness-implementation studies [19], an emerging type of study design that emphasizes both the implementation strategies involved in intervention delivery and the intervention effectiveness. We also incorporated implementation science frameworks and human-centered design methodology in designing delivery strategies that target key barriers to acceptance and completion of 3HP [20], including an emphasis on shared-decision making [21]. Key elements of trial design and execution are designed towards the pragmatic end of the pragmatic-explanatory continuum as described by the PRECIS-2 framework [22]. Finally, we utilized the RE-AIM framework to define outcomes across multiple domains relevant to understanding real-world impact of the three delivery strategies [23].

#### Effectiveness-implementation (EI) hybrid trial design

The overall study design was influenced by hybrid effectiveness-implementation trials, which blend components of clinical effectiveness research and implementation science. By doing so, hybrid trials accelerate the translation of evidence-based findings into routine practice. On the spectrum of hybrid study designs, the *3HP Options Trial* is classified as type 3 [19]: our primary goal is to identify the optimal implementation (i.e., delivery) strategy for 3HP rather than its effectiveness. Thus, the primary outcome reflects the reach (acceptance) and fidelity (treatment completion) to the intervention; assessment of health outcomes (incident TB, adverse events, mortality) is a secondary aim.

#### Behavior Change Wheel framework

We designed our DOT and SAT delivery strategies to target key barriers to 3HP acceptance and completion using the Behavior Change Wheel (BCW) framework, which was developed to address the lack of frameworks that were coherent, comprehensive, and/or grounded in a general theory or model of behavior change [20]. The BCW framework uses the capability opportunity motivation behavior (COM-B) model to understand behavior. COM-B specifies that changing any behavior requires changing capability, opportunity, and/or motivation to perform the behavior. The BCW framework then identifies functions that effective interventions should serve to target barriers within each COM-B domain, and evidence-based behavior change techniques to carry out those functions. Thus, the BCW framework provides a coherent basis for considering potential barriers to behavior change and the intervention components expected to overcome those barriers.

#### Shared decision-making

Shared decision-making (SDM) is an important aspect of patient-centered care, which the Institute of Medicine has identified as one of six key components of high-quality health care [24]. Key characteristics of shared decision-making include (1) information sharing, (2) participation by all parties in consensus building about preferred treatment, and (3) reaching an agreement about treatment [21]. SDM interventions improve knowledge and accuracy of risk perceptions, decisional conflict, match between personal values and choice, and compliance with medical care [25, 26]. SDM interventions are also feasible and more beneficial to disadvantaged groups than to those with higher literacy, education, and socioeconomic status, particularly when tailored to the situation [27]—suggesting that SDM could be very effective in resource-limited settings. An example of a SDM tool is decision aids, which are most useful for decisions in which there is more than one medically reasonable option (e.g., receiving 3HP by DOT vs. SAT), such that the choice between options aligns with the patient’s values and preferences [28].

#### Human-centered design

Human-centered design (HCD) methods were used for the development of two key components of the delivery strategies, including the SDM tool and the 99DOTS pill packaging. HCD aims to develop more usable practices in real-world contexts and involves systematically collecting stakeholder and user input throughout the development and testing process of a product, allowing for various iterations and updates of a product design [29]. Using HCD methods, we conducted formative research with patients and Mulago HIV/AIDS clinic providers to develop, pilot test, and refine a decision aid tool aimed to facilitate a shared decision-making process for patients randomized to choose between DOT and SAT delivery strategies. We also undertook a re-design of the 99DOTS pill packaging, both to better cater towards patients in this context and to also accommodate weekly medication dosing. Applying HCD methods, we tested several iterations of the packaging and incorporated patient and provider feedback on each to finalize a 99DOTS pill package design. Further details on the re-design of the 99DOTS pill package will be reported elsewhere.

#### RE-AIM

The RE-AIM evaluation framework enables trialists to consider outcomes across a number of domains to better understand the sustainable adoption and implementation of evidence-based interventions. The *3HP Options Trial* includes outcomes across the following RE-AIM domains: (1) reach of the three different strategies within the intended target population; (2) effectiveness of the intervention itself and of the strategies in promoting fidelity to the intervention; (3) factors that promote adoption of the delivery strategies by clinic providers; and (4) fidelity, acceptability, and costs of intervention implementation under each strategy.

## Methods/design

### Design overview

The *3HP Options Trial* will be a three-arm, individual participant randomized trial, with a hybrid type 3 effectiveness-implementation design. Eligible and consenting participants will be randomized with equal allocation to one of three optimized strategies for delivery of 3HP treatment with once weekly INH and RPT for 12 weeks: facilitated DOT, facilitated SAT, or an informed choice between facilitated DOT and facilitated SAT (with the assistance of a decision aid tool). Participants randomized to the facilitated DOT arm will attend the Mulago HIV/AIDS clinic on a weekly basis to ingest 3HP medication under direct observation. Those randomized to facilitated SAT will take medication at home and report medication adherence using the 99DOTS platform, with an in-person refill visit at their week 6 dose. Choice participants will decide between the two delivery strategies, with the option to switch between DOT and SAT at any time. Embedded mixed methods and health economic sub-studies will assess the adoption, implementation, and cost/cost-effectiveness of the three delivery strategies.

This study was reviewed and approved by the University of California San Francisco Committee on Human Research, the Makerere University School of Public Health Research Ethics Committee, and the Uganda National Council for Science and Technology. The protocol is registered on ClinicalTrials.gov (NCT03934931) as a phase 4 clinical trial and complies with the reporting guidelines outlined in the pragmatic trial extension of the Consolidated Standards of Reporting Trials (CONSORT; see Additional file 1) [30].

### Study setting and population

The trial will take place at the Makerere University Joint AIDS Program (MJAP) Mulago Immune Suppression Syndrome (i.e., HIV/AIDS) clinic, which is the largest outpatient HIV clinic in Uganda (16,000 PLHIV enrolled and 300 new PLHIV registered monthly) and accepts patients from multiple HIV testing sites in Kampala.

**Fig. 1:**
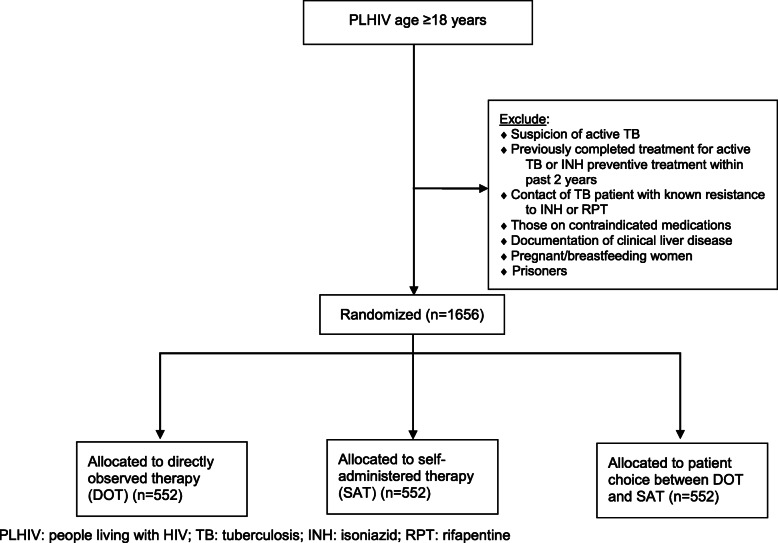
Study flow diagram (CONSORT)

The trial will include patients 18 years and above engaged in routine care at the Mulago HIV/AIDS clinic. Patients will be recruited from the patient waiting area by peer educators who provide routine education including about TB prevention. Interested patients will be referred to study staff for eligibility screening and will be excluded if they meet any of the following routine criteria that are contraindications to 3HP: (1) suspicion of active TB or current/planned TB treatment; (2) actively taking antiretroviral (ARV) medication contraindicated for use with rifapentine; (3) contact with a TB patient with known resistance to isoniazid or rifamycins; (4) women who are pregnant, breastfeeding, or intending to get pregnant within 120 days; (5) previously completed treatment for active TB or at least 6 months of isoniazid preventive therapy within the past 2 years; (6) pre-existing documentation of clinical liver disease or alcoholism; or (7) actively taking any medication contraindicated for use with rifapentine. In addition, patients will be excluded if they (1) are a prisoner, (2) do not intend to remain within 25 km of the Mulago HIV/AIDS clinic during the study period or do not intend to receive further care at the Mulago HIV/AIDS clinic (to enable proper follow-up), (3) do not have access to a mobile telephone or are unwilling to receive reminder phone calls (which would interfere with the SAT delivery strategy), (4) live with another household member currently enrolled in the study, or (5) are not able to provide informed consent in either English or Luganda. We will recruit and screen patients until we meet our sample size of 1656 total patients (*n* = 552/arm; Fig. 1). Planned mixed methods and health economic sub-studies will include a subset of participants enrolled in the trial and Mulago HIV/AIDS clinic staff involved in providing TPT services.

### Randomization

All eligible consenting participants to be included in the study will be randomized in a 1:1 ratio to one of the three arms using permuted block randomization with block sizes between 9 and 15. Randomization numbers will be generated using a statistical software algorithm. Individual random assignment sheets will be placed and sealed in opaque envelopes in batches of multiple blocks. Each eligible participant will select an envelope which s/he will open to see the randomization number and study arm.

### Intervention delivery strategies

**Fig. 2:**
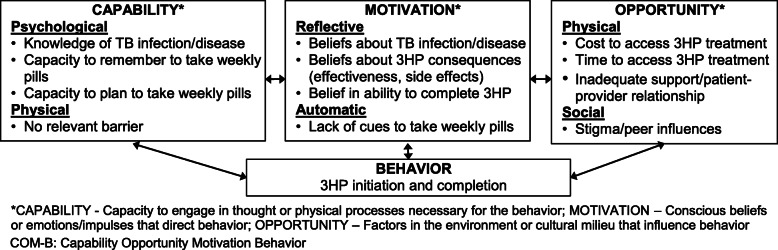
Patient barriers to treatment adherence categorized according to the COM-B model. Critical barriers were identified through stakeholder consultation and targeted in order to facilitate 3HP uptake and adherence via DOT or SAT delivery strategies

Design elements of the three study arms were developed using a theory-informed approach for understanding and targeting potentially modifiable barriers to medication adherence relevant to 3HP treatment at a HIV/AIDS clinic in sub-Saharan Africa. Specifically, using the BCW framework, we worked with local stakeholders in Uganda to first identify patient barriers to medication adherence [31], and then (1) prioritized key barriers to target in order to facilitate 3HP delivery using DOT or SAT; (2) selected intervention functions likely to address each key barrier; (3) selected behavior change techniques likely to help enact each intervention function; and (4) selected a feasible mode of delivery for each behavior change technique (Fig. 2). Applying the COM-B model and working with local stakeholders, we considered critical barriers to 3HP uptake to include the following: (a) Capability—poor understanding of the need for preventive therapy and inadequate memory or planning capacity; (b) Opportunity—long wait times and economic costs required for clinic visits; and (c) Motivation—beliefs about relative harms vs. benefits, lack of self-efficacy in completing 3HP, and lack of cues to take weekly medications. We designed three delivery strategies that contain specific components targeting each of these critical barriers to facilitate treatment completion (Table 1).

**Table 1:** Targeted barriers and content of 3HP delivery strategies

Capability			Opportunity			Motivation			Selected intervention function(s)	Selected behavior change technique	Selected mode of delivery
Knowledge	Capacity to remember	Capacity to plan	Time to access 3HP	Cost to access 3HP	Inadequate support	Beliefs about consequences	Self-efficacy	Lack of cues/reminders			
**Facilitated directly-observed therapy (DOT)**											
✓						✓			Education, persuasion	Information about health consequences	Standardized counseling by clinic nurse
			✓	✓					Environmental restructuring	Restructuring physical environment	Streamlined weekly clinic visits + cost reimbursement
	✓							✓	Enablement	Prompts/cues	Appointment reminders via Interactive Voice Response (IVR)
✓					✓	✓	✓		Education, persuasion	Credible source	Weekly visit with health worker (DOT)
**Facilitated self-administered therapy (SAT)**											
✓						✓			Education, persuasion	Information about health consequences	Standardized counseling by clinic nurse
			✓	✓					Environmental restructuring	Restructuring physical environment	SAT + streamlined monthly clinic visits + cost reimbursement
	✓	✓						✓	Enablement	Prompts/cues	Dosing reminders and confirmation via IVR
					✓		✓		Enablement	Social support	Weekly check-in via IVR
**Patient choice between facilitated DOT and facilitated SAT**											
✓					✓	✓	✓		Education, enablement	Problem solving	Shared decision-making, facilitated by decision aid

#### Arm 1: facilitated DOT

Participants randomized to the facilitated DOT arm will attend the Mulago HIV/AIDS clinic on a weekly basis to ingest 3HP medication under direct observation. In particular, participants randomized to facilitated DOT will receive the following:

1. Streamlined clinic visits: DOT cards with instructions to present directly to the pharmacy for a pharmacy-only visit, without the need to wait in the general queue.
2. Cost reimbursements: A fixed level of reimbursement (15,000 Ugandan Shillings; ~ $5 US Dollars/visit) for each weekly visit, conditional on either directly observed therapy or evidence of an adverse event that would preclude further treatment.
3. Appointment reminders: Automated IVR phone call reminders (at no cost to participants) the day before each appointment.

By minimizing the time and costs of accessing 3HP while also promoting adherence through regular reminders, the facilitated DOT arm was designed to address barriers to capability, opportunity, and motivation of accessing and adhering to 3HP (Table 1).

#### Arm 2: facilitated SAT

Participants randomized to the facilitated SAT arm will take their first dose of medication under direct observation and be provided with a 4-week supply of 3HP to take weekly via self-administration (doses two, three, four, and five). Weekly doses will be pre-sorted in the re-designed 99DOTS pill package with a card insert for each dose displaying a phone number used to confirm dosing. Participants will be asked to return to the Mulago HIV/AIDS clinic after completing their fifth dose to review adherence data with a clinic pharmacy technician and receive five additional doses of 3HP (doses 7–11). At the scheduled refill visit (dose 6) and the end-of-treatment visit (dose 12), participants will ingest 3HP doses under direct observation by a pharmacy technician. Similar to the facilitated DOT arm, the following design components of facilitated SAT were included to address barriers to capability, opportunity, and motivation (Table 1):

1. Cost reimbursements: A fixed level of reimbursement (same as facilitated DOT) for the refill and end-of-treatment visit, conditional on either directly observed therapy or evidence of an adverse event that would preclude further treatment.
2. Dosage reminders: Automated weekly dosing reminder phone call via the 99DOTS-based platform to promote adherence.
3. Weekly check-ins: Two-way IVR phone calls via the 99DOTS platform asking, “Are you well?” (or other similar message, as determined in our formative research phase), to serve as an alternative to weekly visits with a health worker to inquire about potential side effects.

#### Arm 3: patient choice between facilitated DOT and facilitated SAT

Participants randomized to patient choice between facilitated DOT and facilitated SAT arm will be offered an informed choice between the two facilitated delivery strategies. A research nurse will use the decision aid designed for this context using HCD methods to engage the patient in a discussion regarding his or her values and preferences, and, after addressing any questions, ask the participant to select facilitated DOT or facilitated SAT. By offering patients an informed choice between two optimized delivery strategies and supporting their decision-making process using a patient-centered approach, we effectively target capability, opportunity, and motivational barriers to 3HP adherence (Table 1).

### Blinding

Given the nature of the delivery strategies, blinding participants and health care providers to the assigned study arm will not be possible. Aggregated patient-level data will be prepared by the study statistician and data manager, who will present data masked by study arm to all other investigators and study staff. An independent Steering Committee will assist in monitoring data collection, study progress, and patient safety.

### Assessments and data collection

Along the spectrum of explanatory to pragmatic [22], we aimed for most aspects of our trial design to reflect usual care as much as possible by implementing intervention delivery and patient monitoring and follow-up through routine clinic staff (see Additional file 2). Thus, to facilitate evaluation of 3HP delivery under usual clinic conditions, research staff will not interact with participants between treatment initiation and either discontinuation or completion. Mulago HIV/AIDS clinic staff will perform all activities related to 3HP treatment during DOT or SAT refill visits, including screening participants for side effects, screening participants for active TB, dispensing 3HP medicines, and, for participants taking 3HP by SAT, reviewing electronic dosing records and responses to weekly IVR phone call check-ins in the 99DOTS platform. Research staff will be responsible for abstracting routine patient data from paper and electronic medical records (including the 99DOTS server), and conducting research activities including participant consent, randomization, and baseline and exit questionnaires. In addition, research staff will survey and interview a small subset of participants for implementation and health economic sub-studies during and after 3HP treatment. All patient data will be entered into Research Electronic Data Capture (REDCap), a secure web-based application used for data collection and management in clinical research [32, 33].

**Fig. 3:**
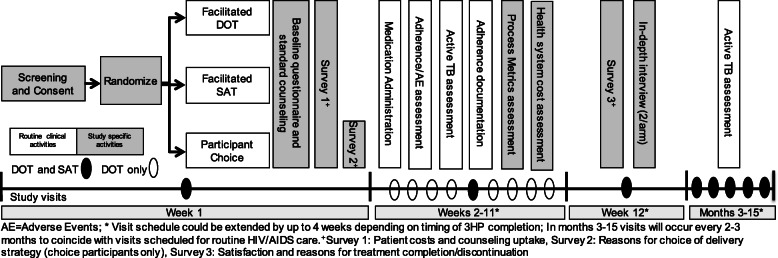
*3HP Options Trial* study procedures and timeline. Patients are screened, consented, randomized, and followed up for up to 15 months following study enrollment. Study staff facilitate all enrollment procedures at baseline, intermittent surveys, and interviews with selected patients during 3HP treatment and the end-of-study visit at 15 months. Patient interactions during and following 3HP treatment including 3HP dosing and monitoring for side effects are otherwise managed by routine Mulago HIV/AIDS clinic staff through the end of patient follow-up

The timing of the following data collection activities informing study outcomes is outlined in Fig. 3:

A. Baseline assessment: The baseline questionnaire will collect participant demographic information, socioeconomic data, and medical history.
B. Weekly adherence assessments: 3HP dosing will be assessed using weekly adherence data abstracted from the 99DOTS server and/or the 3HP medication administration log.
C. Patient surveys and in-depth interviews: Focused data collection activities including surveys and in-depth interviews with patients after study exit (regardless of completion of 3HP) will assess satisfaction with and acceptability of each delivery strategy, identify processes and contextual factors that support or hinder 3HP acceptance and completion, and ascertain whether delivery strategy components successfully modified targeted barriers. Patients in the choice arm will complete a survey including the validated Shared Decision-Making Questionnaire (SDM-Q-9) to assess the implementation of the shared decision-making process [34, 35]. In addition, patient costing surveys conducted at the beginning of 3HP delivery supplemented by a second costing survey at 6 weeks into treatment will assess the direct and indirect costs of TPT (e.g., ancillary healthcare visits, patient and caregiver costs associated with 3HP, use of formal social support programs). Surveys are based on the WHO handbook for TB patient costing surveys, which includes family and coping costs, and which we have adapted and used in Uganda [36].
D. Provider interviews: Key informant interviews with health service providers at the conclusion of the study period will be used to assess acceptability and clinic-level facilitators and barriers to adoption and implementation of 3HP under each delivery strategy.
E. Process metric data: Process metric data will be collected periodically throughout the course of the study and will be used to monitor adoption of and fidelity to components of the delivery strategies. This includes an assessment of if and when participants received reimbursement for clinic visits during the course of 3HP treatment, and data collected from the 99DOTS server to understand (1) the proportion of self-administered doses confirmed by phone calls, (2) the proportion of outgoing appointment/dosing reminders and check-in IVR phone calls delivered, and (3) the proportion of incoming SMS messages (response to check-in) received or IVR phone calls answered.
F. Health economic evaluation: Health economic data will be used to assess costs and cost-effectiveness of intervention implementation under each delivery strategy. Empirical costing data collection activities will include (1) a detailed budgetary analysis involving interviews with key staff members; review of logbooks/timesheets; review of the space, cost, and time spent on SDM activities; and review of 99DOTS implementation costs; and (2) time-and-motion studies to assess clinic staff involved in TPT delivery and patient time and resources required for visits specific to DOT or SAT.
G. Incident TB**:** Patients will be screened for a period of 1 year after 3HP treatment completion (i.e., up to 16 months after enrollment) at routine HIV/AIDS clinic visits. TB screening will include a symptom assessment using the WHO four-symptom TB screen administered at every visit and for patients with new or progressive TB symptoms a chest X-ray and sputum for Xpert MTB/RIF and mycobacterial culture, as per routine WHO and National TB and Leprosy Programme recommended procedures. All participants will have sputum collected at their last study visit (15–16 months after enrollment) for TB culture.

## Analysis

### Outcomes

Outcomes were developed in accordance with domains of the RE-AIM evaluation framework. The primary outcome was conceptualized in order to assess both intervention reach (acceptance) and fidelity (treatment completion) under each delivery strategy and is defined as the proportion of patients who accept and complete at least 11 of the 12 weekly doses of 3HP within 16 weeks of study enrollment. Other trial outcomes, categorized within appropriate RE-AIM domains, are outlined in Table 2.

**Table 2:** Trial secondary outcomes categorized using the RE-AIM framework

RE-AIM Domain	Outcome
**Reach**	• Proportion of eligible PLHIV offered 3HP who accept to initiate treatment
**Effectiveness/cost-effectiveness**	**Effectiveness of intervention** • Proportion of participants who initiate 3HP for whom treatment is discontinued due to adverse events or intolerance • Cumulative 16-month incidence of active TB **Cost-effectiveness** • Incremental cost of each delivery strategy per disability adjusted life year (**DALY**) averted • Incremental health system cost per DALY averted • Incremental patient cost per DALY averted
**Adoption**	• Thematic results from healthcare provider key informant interviews
**Implementation**	**Fidelity of intervention and delivery strategy implementation** • Proportion who complete at least 11 of 12 3HP doses within 16 weeks of starting treatment • Proportion reimbursed overall and on the same day as each 3HP clinic visit • Time spent at each clinic visit • Time spent on shared decision-making tool • Proportion of IVR phone call reminders delivered to participants for clinic visits or medication dosing • Proportion of IVR phone call reminders delivered to participants for missed appointments • Proportion of participants screened for active TB during DOT or refill visits • Proportion of participants screened for side effects during DOT or refill visits. • Proportion of doses confirmed using digital adherence technology. Doses directly observed (i.e., during initial or refill visits) will not be included in the denominator^a^ • Proportion of IVR phone call reminders delivered to participants following missed doses^a^ • Proportion of weekly IVR phone call check-ins delivered to participants^a^ • Proportion of responses to weekly IVR phone call check-ins received from participants^a^ • Proportion of participants who receive appropriate follow-up (phone call or home visit) for lack of response/negative response to weekly check-in IVR phone call^a^ **Patient costs** • Total direct and indirect patient costs related to TB preventive care services **Acceptability of delivery strategy** • Median scores for domains within the patient satisfaction survey • Median scores for domains within the SDM-Q-9 validated questionnaire on implementation of the shared decision-making tool **Modification of targeted barriers** • Self-reported patient barriers to TB preventative care services • Patient satisfaction with TB preventive care services • Provider- and clinic-level barriers to delivery of 3HP • Thematic results from patient in-depth interviews

^a^Participants taking 3HP by SAT only

### Study hypothesis and primary analysis

The primary study hypotheses are that (1) the proportion of PLHIV who accept and complete 3HP can exceed 80% in a high HIV/TB burden setting and (2) the proportion who accept and complete 3HP will be highest among PLHIV randomized to the informed choice arm. Primary outcome analysis will include (1) simple calculation (using the exact binomial confidence intervals) of the effectiveness of each delivery option, with a target of 80% and (2) unadjusted intent-to-treat comparisons of effectiveness between arms. In these analyses, participants randomized to the informed choice arm will be treated separately from those randomized to either facilitated DOT or facilitated SAT; the proportion of those in the patient choice arm who choose DOT or SAT will be measured, but the primary outcome will consider all individuals in this arm as randomized to patient choice. Whether 80% acceptance and completion is achieved will be based on the lower bound of the Bonferroni-corrected 97.5% confidence interval exceeding 0.80 in any of the three arms. The intent-to-treat comparisons will use log-binomial regression to calculate prevalence ratios and Fisher’s exact test to determine statistical significance (two-sided alpha of 0.025, including Bonferroni correction). Participants who decline to initiate 3HP after randomization will be counted as not accepting/completing treatment in the primary (intent-to-treat) analysis and will be followed over time for clinical outcomes.

### Sample size and power

We estimated our sample size based on a minimum clinically important difference of 10% in 3HP completion, comparing patient choice vs. DOT arms. Of note, a similar study of 3HP delivery strategies chose a 15% non-inferiority margin between DOT and SAT based on cost-effectiveness modeling in the USA [15]. We chose 10% to be more conservative because similar modeling studies have not been done in low-income settings and because ours is not a non-inferiority design. To be maximally conservative, we applied a Bonferroni correction based on two independent comparisons (choice vs. DOT and choice vs. SAT). Assuming a two-sided alpha of 0.025 and 5% loss between consent and allocation, a sample size of 552 participants per arm (1656 total) is required to provide power of 0.90 to detect this difference. This sample size will also give us power of 0.85 to detect a point estimate of at least 80% effectiveness in the patient choice arm, assuming a true effectiveness of 85%. If the true effectiveness rises to 86%, our power to show effectiveness > 80% increases to 0.96.

## Discussion

In order to realize the promising potential of 3HP and reduce the burden of TB among PLHIV, there is a crucial need for trials that incorporate theory-informed approaches to medication delivery that are optimized to facilitate treatment adherence and completion. However, to date, the only trial assessing strategies for 3HP delivery in a high-burden setting was (1) not optimized to facilitate treatment completion for either DOT or SAT and (2) did not consider patient preference for one delivery strategy over another—despite strong reasons for patients to prefer SAT vs. DOT or vice versa. Not surprisingly, high levels of treatment completion across all settings were not achieved, particularly for SAT in high burden settings [15]. With the *3HP Options Trial*, we propose a pragmatic three-armed randomized study in order to generate high-quality evidence demonstrating that if delivery strategies are designed to address patient barriers, high levels of 3HP treatment completion can be achieved in the context of routine HIV/AIDS care in sub-Saharan Africa.

Hybrid trials have a dual focus on assessing implementation and effectiveness outcomes, enabling accelerated translation of evidence-based findings into routine practice relative to the traditional step-wise pipeline model [37]. As a hybrid type 3 study design, the *3HP Options Trial* primarily aims to test implementation strategies for 3HP, while also adding to the strong existing evidence base demonstrating its safety and effectiveness on health outcomes [9–11, 38]. We incorporated elements of pragmatic research into our study design, primarily by emulating how the delivery strategies would be implemented in usual care conditions wherever possible; this included clinic staff performing all activities related to 3HP dosing, adverse events monitoring, and adherence monitoring during the course of 3HP treatment. By taking this innovative yet pragmatic approach to our research design, we anticipate that results will establish what is possible in routine settings, maximize translational gains, and promote ease of potential future scale-up efforts in Uganda and beyond.

This trial is innovative in several other ways. We incorporated implementation science conceptual frameworks, novel digital technologies, and principles from human-centered design into components of the three delivery strategies. Among these, we highlight shared decision-making as a unique and key element of our study design. By promoting self-efficacy with supportive decision making [21, 39], we anticipate that offering patients an informed choice regarding their care will lead to greater treatment acceptance and adherence. To the best of our knowledge, this will be the first trial to incorporate a patient-centered strategy for delivery of TPT to PLHIV in a high-burden, low-resourced setting. In addition, the trial will contribute to the sparse literature on SDM interventions, including the individual- and organizational-level factors that may influence uptake, in the global health context [40]. Thus, the trial will inform how SDM interventions for preference sensitive healthcare decisions can be developed and implemented in low-income countries.

To summarize, we describe a type 3 hybrid effectiveness implementation randomized controlled trial that aims to understand the best facilitated strategy for delivering short-course TB preventive therapy to PLHIV in Uganda. We feature SDM as a crucial component of our study design, contributing to the limited number of studies implementing SDM interventions in low-income countries such as Uganda [40], and propose a comparison of three different delivery strategies that each address capability, opportunity, and motivation barriers to treatment completion. We anticipate that by fulfilling the main study objectives we will provide a comprehensive comparative assessment of these patient-centered approaches to delivery of 3HP via DOT or SAT, including patient choice between the two strategies. The results will enable program officials and policymakers to better deliver this critical advance in TB prevention to PLHIV, who still die of TB more often than from any other cause.

## Supplementary information

**Additional file 1.** Checklist of items for reporting pragmatic trials

**Additional file 2. **PRECIS-2 scores for *3HP Options Trial* domains

## Data Availability

Not applicable.

## Abbreviations

ARV: Antiretroviral
BCW: Behavior Change Wheel
COM-B: Capability opportunity motivation behavior
CONSORT: Consolidated Standards of Reporting Trials
DOT: Directly observed therapy
EI: Effectiveness-implementation
INH: Isoniazid
ISS: Immune suppression syndrome
IVR: Interactive voice response
LMIC: Low- and middle-income countries
MJAP: Makerere Joint AIDS Program
PLHIV: People living with HIV
REDCap: Research Electronic Data Capture
RPT: Rifapentine
SAT: Self-administered therapy
SDM: Shared decision-making
SDM-Q-9: Shared Decision-Making Questionnaire
TB: Tuberculosis
TPT: Tuberculosis preventive therapy
WHO: World Health Organization
