# Supplementary material for: Protocol for the 3HP Options Trial: a hybrid type 3 implementation-effectiveness randomized trial of delivery strategies for short-course tuberculosis preventive therapy among people living with HIV in Uganda
Source: Implement Sci. 2020 Aug 12;15:65. doi: 10.1186/s13012-020-01025-8 (PMC7425004; doi:10.1186/s13012-020-01025-8)
Supplement: Supplementary file 2 — Additional file 2. PRECIS-2 scores for 3HP Options Trial domains [file 13012_2020_1025_MOESM2_ESM.docx]

**Additional file 2. PRECIS-2 scores for *3HP Options Trial* domains.**

| **PRECIS-2 Domain** | **Score** | **Rationale** |
| --- | --- | --- |
| 1. Eligibility | 4 | The study population is adults enrolled in HIV/AIDS care at the Mulago HIV/AIDS clinic who do not have a contraindication for 3HP. Only prisoners (unable to make an independent informed choice of delivery strategy), those who do not intent do stay within 25 kilometers of the clinic (low likelihood of completing treatment by DOT), those who do not have access to a mobile phone (unable to receive the SAT delivery strategy), and either live with another household member enrolled in the study or are unable to provide informed consent (feasibility reasons) are excluded from the target study population. |
| 2. Recruitment | 5 | Patients who come to the clinic for routine appointments will be recruited from the patient waiting area by peer educators who provide routine education including about TB prevention. Interested patients will be referred to study staff for eligibility screening. There are no other targeted recruitment efforts. |
| 3. Setting | 3 | Single center study at a large, urban HIV/AIDS clinic. |
| 4. Organization | 4 | The study facilitated the hiring of one additional clinic pharmacist to help with 3HP delivery in addition to routine clinic activities. There are no other changes to the organizational setting. |
| 5. Flexibility (delivery) | 5 | Routine clinic staff will perform all activities related to 3HP treatment during DOT and SAT refill visits, including screening for side effects and active TB, dispensing 3HP medicines, monitoring 3HP adherence, and making decisions regarding patient care. |
| 6. Flexibility (adherence) | 5 | Other than consenting patients into the *3HP Options Trial*, no procedures or interventions related to adherence are included beyond what would occur if the proposed delivery strategies were implemented in usual care. |
| 7. Follow-up | 5 | No additional study-specific procedures or follow-up are included during the period of 3HP treatment with the exception of surveys and interviews with a small subset of patients. Patients are passively followed up for up to 1 year following completion of 3HP treatment by extracting data from their routinely scheduled HIV/AIDS clinic appointments with Mulago clinic staff. |
| 8. Primary outcome | 5 | Acceptance and completion of a preventive treatment reflects patients’ beliefs about and experience of benefits vs. side effects, inconvenience and costs. |
| 9. Primary analysis | 5 | All patients randomized will be included in the analysis, regardless of whether they choose to initiate or complete 3HP treatment. |
